# Supplementary material for: The Six-Item Version of the Internet Addiction Test: Its Development, Psychometric Properties, and Measurement Invariance among Women with Eating Disorders and Healthy School and University Students
Source: Int J Environ Res Public Health. 2021 Nov 24;18(23):12341. doi: 10.3390/ijerph182312341 (PMC8657305; doi:10.3390/ijerph182312341)
Supplement: Supplementary file 1 [file ijerph-18-12341-s001.zip › Supplementary Appendix 1. The six-item Internet Addiction Test.pdf]

Items of the six-item Internet Addiction test along with the corresponding addictive domains.

**Addictive domains**	**Items**
Conflict	6. Does your work suffer (e.g., postponing things, not meeting deadlines, e tc.) because of the amount of time you spend online?
Mood modification	10. Do you block disturbing thoughts about your life with soothing thoughts of the Internet?
Salience	15. Do you feel preoccupied with the Internet when offline or fantasize about being online?
Tolerance	16. Do you find yourself saying “Just a few more minutes” when online?
Relapse	17. Do you try to cut down the amount of time you spend online and fail?
Withdrawal	20. Do you feel depressed, moody, or nervous when you are offline, which goes away once you are back online?
