# Supplementary material for: The Six-Item Version of the Internet Addiction Test: Its Development, Psychometric Properties, and Measurement Invariance among Women with Eating Disorders and Healthy School and University Students
Source: Int J Environ Res Public Health. 2021 Nov 24;18(23):12341. doi: 10.3390/ijerph182312341 (PMC8657305; doi:10.3390/ijerph182312341)
Supplement: Supplementary file 1 [file ijerph-18-12341-s001.zip › Supplementary Appendix 2. The Spanish version of the Internet Addiction Test.pdf]

The Spanish version of the Internet Addiction Test

Item	Pregunta
1	¿Con qué frecuencia te da la impresión de que pasas más tiempo en internet del que pensabas?
2	¿Con qué frecuencia dejas las labores del hogar por estar conectado a internet?
3	¿Con qué frecuencia prefieres la emoción que te proporciona internet a intimar con tu pareja?
4	¿Con qué frecuencia entabla nuevas amistades con otros usuarios de internet?
5	¿Con qué frecuencia protesta la gente de tu entorno por el tiempo que pasas en internet?
6	¿Con qué frecuencia se ve perjudicado tu rendimiento académico por el tiempo pasado en internet?
7	¿Con qué frecuencia miras tu correo antes de hacer otra cosa que tienes que hacer?
8	¿Con qué frecuencia se ve perjudicado tu rendimiento o productividad en el trabajo por culpa de internet?
9	¿Con qué frecuencia te vuelves reservado o te pones a la defensiva cuando alguien te pregunta lo que haces en internet?
10	¿Con qué frecuencia enmascaras tus problemas de la vida real con pensamientos relajantes sobre internet?
11	¿Con qué frecuencia te ves pensando en el momento en que vas a meterte en internet?
12	¿Con qué frecuencia temes que tu vida sin internet sería aburrida, vacía y carente de felicidad?
13	¿Con qué frecuencia saltas, voceas o te enfadas cuando alguien te molesta mientras estás en internet?
14	¿Con qué frecuencia pierdes horas de sueño porque te quedas hasta altas horas en internet?
15	¿Con qué frecuencia te obsesionas con internet cuando no estás conectado?
16	¿Con qué frecuencia piensas "solo unos minutos más" cuando estás conectado?
17	¿Con qué frecuencia intentas reducir el tiempo que pasas conectado sin conseguirlo?
18	¿Con qué frecuencia intentas ocultar el tiempo que pasas conectado?
19	¿Con qué frecuencia decides seguir en internet en lugar de salir con gente?
20	¿Con qué frecuencia te sientes deprimido, de mal humor o nervioso cuando no estás conectado y se te pasa todo en cuanto vuelves a conectarte?

Escala: 0 = Nunca; 1 = Casi nunca; 2 = Ocasionalmente; 3 = Frecuentemente; 4 = A menudo; 5 = Siempre.

** Reproduced with permission from Fernández-Villa et al [1], BMC Public Health, 2015, http://creativecommons.org/publicdomain/zero/1.0/

1. Fernández-Villa, T.; Molina, A.J.; García-Martín, M.; Llorca, J.; Delgado-Rodríguez, M.; Martín, V. Validation and psychometric analysis of the Internet Addiction Test in Spanish among college students. *BMC Public Health* **2015**, *15*, 953, doi:10.1186/s12889-015-2281-5.
