# Supplementary material for: The Six-Item Version of the Internet Addiction Test: Its Development, Psychometric Properties, and Measurement Invariance among Women with Eating Disorders and Healthy School and University Students
Source: Int J Environ Res Public Health. 2021 Nov 24;18(23):12341. doi: 10.3390/ijerph182312341 (PMC8657305; doi:10.3390/ijerph182312341)
Supplement: Supplementary file 1 [file ijerph-18-12341-s001.zip › Supplementary Table S1.pdf]

Supplementary Table S1. Goodness-of-fit indices for different models of the Internet Addiction Test structure tested in confirmatory factor analysis

**Models**	**Samples**	**χ^2**	***p***	***df***	**CFI**	**TLI**	**RMSEA**	**RMSEA 90% CI**	**SRMR**
Model 1 (C)	Whole sample	654.259	0.001	170	0.886	0.873	0.153	0.141 to 0.165	0.0275
1F 20 items	Group 1	462.130	0.001	170	0.818	0.787	0.172	0.153 to 0.191	0.0494
	Group 2	438.790	0.001	170	0.895	0.882	0.158	0.140 to 0.177	0.0240
Model 2 (E)	Whole sample	511.792	0.001	166	**0.919**	**0.907**	0.131	0.118 to 0.144	0.0252
1F 20 items	Group 1	392.766	0.001	166	**0.859**	**0.838**	0.153	0.134 to 0.173	0.0461
	Group 2	367.182	0.001	166	**0.921**	**0.910**	0.139	0.120 to 0.158	0.0226
Model 3 (C)	Whole sample	563.213	0.001	152	0.897	0.884	0.149	0.136 to 0.162	0.0266
1F 19 items	Group 1	374.480	0.001	152	0.849	0.831	0.159	0.139 to 0.179	0.0457
	Group 2	388.993	0.001	152	0.901	0.889	0.157	0.138 to 0.177	0.0240
Model 4 (E)	Whole sample	491.462	0.001	150	0.915	0.903	0.137	0.123 to 0.150	0.0252
1F 19 items	Group 1	337.925	0.001	150	0.873	0.855	0.147	0.126 to 0.168	0.0438
	Group 2	352.363	0.001	150	0.916	0.904	0.146	0.127 to 0.166	0.0231
Model 5 (C)	Whole sample	634.629	0.001	169	0.891	0.877	0.150	0.138 to 0.163	0.0276
2F 20 items	Group 1	455.022	0.001	169	0.822	0.800	0.172	0.152 to 0.190	0.0495
	Group 2	426.691	0.001	169	0.899	0.887	0.156	0.137 to 0.174	0.0242
Model 6 (E)	Whole sample	464.874	0.001	164	0.929	0.918	0.123	0.110 to 0.136	0.0245
2F 20 items	Group 1	370.324	0.001	164	0.871	0.851	0.147	0.127 to 0.167	.0456
	Group 2	342.935	0.001	164	0.930	0.919	0.132	0.112 to 0.115	0.0221
Model 7 (C)	Whole sample	545.697	0.001	151	0.901	0.888	0.146	0.133 to 0.160	0.0269
2F 19 items	Group 1	367.160	0.001	151	0.854	0.834	0.157	0.137 to 0.178	0.0462
	Group 2	378.530	0.001	151	0.905	0.893	0.155	0.135 to 0.174	0.0243
Model 8 (E)	Whole sample	386.535	0.001	146	**0.940**	**0.930**	0.116	0.102 to 0.130	0.0234
2F 19 items	Group 1	289.827	0.001	146	**0.903**	**0.886**	0.130	0.108 to 0.152	0.0412
	Group 2	297.302	0.001	146	**0.937**	**0.926**	0.128	0.107 to 0.149	0.0219
Model 9 (C)	Whole sample	651.049	0.001	169	0.887	0.873	0.151	0.141to 0.165	0.0272
2F 20 items	Group 1	458.486	0.001	169	0.820	0.797	0.172	0.153 to 0.191	0.0492
	Group 2	438.790	0.001	169	0.894	0.881	0.159	0.141 to 0.178	0.0240
Model 10 (E)	Whole sample	477.796	0.001	164	0.926	0.915	0.125	0.112 to 0.138	0.0245
2F 20 items	Group 1	372.203	0.001	164	0.870	0.85	0.148	0.128 to 0.168	0.0456
	Group 2	351.567	0.001	164	0.927	0.915	0.135	0.115 to 0.154	0.0222
Model 11	Whole sample	398.821	0.001	146	**0.937**	**0.926**	0.119	0.105 to 0.133	0.0235
2F 19 items (E)	Group 1	292.903	0.001	146	**0.901**	**0.884**	0.132	0.110 to 0.154	0.0412

	Group 2	303.875	0.001	146	**0.934**	**0.923**	0.131	0.110 to 0.152	0.0220
Model 12 (C) 3F 20 items	Whole sample	634.232	0.001	167	0.890	0.875	0.151	0.139 to 0.1 64	0.0285
	Group 1	453.495	0.001	167	0.821	0.797	0.172	0.152 to 0.191	0.0500
	Group 2	427.170	0.001	167	0.898	0.884	0.157	0.139 to 0.176	0.0253
Model 13 (E) 3F 20 items	Whole sample	502.340	0.001	163	0.920	0.907	0.131	0.118 to 0.144	0.0265
	Group 1	388.884	0.001	163	0.859	0.836	0.155	0.135 to 0.174	0.0476
	Group 2	381.009	0.001	163	0.915	0.903	0.144	0.125 to 0.163	0.0230
Model 14 (E) 3F 19 items	Whole sample	424.110	0.001	145	0.930	0.918	0.126	0.112 to 0.144	0.0252
	Group 1	308.628	0.001	145	0.889	0.869	0.139	0.118 to 0.161	0.0430
	Group 2	310.115	0.001	145	0.931	0.919	0.134	0.114 to 0.155	0.0235
Model 15 (E) A bifactor 19 items	Whole sample	390.520	0.001	145	**0.939**	**0.928**	0.118	0.104 to 0.132	0.0300
	Group 1	277.055	0.001	145	**0.911**	**0.895**	0.125	0.103 to 0.148	0.0534
	Group 2	281.975	0.001	145	**0.943**	**0.933**	0.122	0.101 to 0.144	0.0205
Model 16 (C) 2F 12 items	Whole sample	224.174	0.001	53	0.928	0.910	0.163	0.141 to 0.185	0.0251
	Group 1	149.886	0.001	53	0.887	0.859	0.178	0.144 to 0.211	0.0454
	Group 2	163.270	0.001	53	0.924	0.905	0.182	0.150 to 0.214	0.0246
Model 17 (E) 2F 12 items	Whole sample	119.646	0.001	49	**0.970**	**0.960**	0.109	0.084 to 0.134	0.0212
	Group 1	90.991	0.001	49	**0.951**	**0.934**	0.122	0.082 to 0.160	0.0433
	Group 2	116.677	0.001	49	**0.953**	**0.937**	0.148	0.114 to 0.183	0.0201
Model 18 (C) 1F six items	Whole sample	29.014	0.001	9	**0.981**	**0.968**	0.135	0.082 to 0.191	0.0189
	Group 1	22.941	0.006	9	**0.962**	**0.936**	0.163	0.082 to 0.284	0.0443
	Group 2	19.650	0.020	9	**0.983**	**0.972**	0.137	0.052 to 0.220	0.0140
Model 19 (E) 1F six items	Whole sample	**8.695**	**0.275**	7	**0.998**	**0.996**	**0.045**	0.000 to 0.126	**0.0096**
	Group 1	**8.748**	**0.271**	7	**0.995**	**0.990**	**0.066**	0.000 to 0.183	**0.0289**
	Group 2	**7.441**	**0.384**	7	**0.999**	**0.998**	**0.032**	0.000 to 0.160	**0.0086**

χ^2: chi-square; df: degrees of freedom; CFI: comparative fit index; TLI: Tucker–Lewis index; RMSEA: root mean square error of approximation; CI: confidence interval; SRMR: standardized root mean residual; F: factor; (C): crude model; (E): the model involves correlating residuals. Values in bold denote acceptable/good fit.
