# Supplementary material for: The Six-Item Version of the Internet Addiction Test: Its Development, Psychometric Properties, and Measurement Invariance among Women with Eating Disorders and Healthy School and University Students
Source: Int J Environ Res Public Health. 2021 Nov 24;18(23):12341. doi: 10.3390/ijerph182312341 (PMC8657305; doi:10.3390/ijerph182312341)
Supplement: Supplementary file 1 [file ijerph-18-12341-s001.zip › Supplementary Table S2.pdf]

Supplementary Table S2. Invariance of 12-item IAT across different student groups

Groups	Invariance levels	χ^2	df	*P*	$\Delta\chi^2$	Δdf	$p(\Delta\chi^2)$	CFI	ΔCFI	TLI	ΔTLI	RMSEA	ΔRMSEA	SRMR
Gender	Configural	386.503	100	0.001				.934		.913		0.051		0.0466
	Metric	394.771	110	0.001	8.268	10	0.603	.935	-0.001	.922	-0.002	.048	0.019	0.0473
	Strong	398.032	113	0.001	3.261	3	0.353	.935	0.004	.924	0.007	.048	-0.038	0.0484
	Strict	485.964	128	0.001	87.932	15	0.001	.918	0.000	.916	-0.002	.050	0.006	0.0488
Nationality	Configural	384.084	100	0.001				.932		.910		0.052		0.0431
	Metric	392.354	110	0.001	8.270	10	0.602	.932	-0.001	.919	-0.002	.050	0.019	0.0480
	Strong	403.478	113	0.001	11.124	3	0.011	.930	0.004	.919	0.007	.050	-0.038	0.0494
	Strict	429.452	128	0.001	24.973	15	0.038	.928	0.000	.925	-0.002	.048	0.006	0.0503
Major	Configural	582.604	256	0.001				.926		.924		0.034		0.0854
	Metric	589.774	266	0.001	7.170	10	0.709	.927	-0.001	.927	-0.002	.033	0.019	0.0847
	Strong	591.661	269	0.001	1.887	3	0.596	.927	0.004	.928	0.007	.033	-0.038	0.0846
	Strict	617.909	284	0.001	26.248	15	0.035	.924	0.000	.930	-0.002	.032	0.006	0.0880
Academic Grade	Configural	597.208	256	0.001				.922		.920		0.035		0.0756
	Metric	601.176	266	0.001	3.968	10	0.949	.924	-0.001	.924	-0.002	.034	0.019	0.0756
	Strong	602.580	269	0.001	1.404	3	0.705	.924	0.004	.925	0.007	.033	-0.038	0.0752
	Strict	616.086	284	0.001	13.506	15	0.563	.924	0.000	.930	-0.002	.032	0.006	0.0766
Internet activities	Configural	620.787	256	0.001				.916		.913		0.036		0.0860
	Metric	634.746	266	0.001	13.959	10	0.175	.915	-0.001	.916	-0.002	.035	0.019	0.0889
	Strong	635.420	269	0.001	0.674	3	0.879	.916	0.004	.917	0.007	.035	-0.038	0.0848
	Strict	660.831	284	0.001	25.411	15	0.045	.913	0.000	.919	-0.002	.034	0.006	0.0910

χ^2: chi-square; df: degrees of freedom; CFI: comparative fit index; TLI: Tucker–Lewis index; RMSEA: root mean square error of approximation; CI: confidence interval; SRMR: standardized root mean residual.
