# Supplementary material for: The Six-Item Version of the Internet Addiction Test: Its Development, Psychometric Properties, and Measurement Invariance among Women with Eating Disorders and Healthy School and University Students
Source: Int J Environ Res Public Health. 2021 Nov 24;18(23):12341. doi: 10.3390/ijerph182312341 (PMC8657305; doi:10.3390/ijerph182312341)
Supplement: Supplementary file 1 [file ijerph-18-12341-s001.zip › Supplementary Table S3.pdf]

Supplementary Table S3. Invariance of six-item IAT across different student groups

Groups	Invariance levels	χ^2	df	*P*	$\Delta\chi^2$	Δdf	$p(\Delta\chi^2)$	CFI	ΔCFI	TLI	ΔTLI	RMSEA	ΔRMSEA	SRMR
Gender	Configural	43.324	14	0.001				.980		.958		0.043		0.0225
	Metric	45.149	19	0.001	1.826	5	0.873	.982	-0.001	.972	-0.002	.035	0.019	0.0223
	Strong	48.583	20	0.001	3.434	1	0.064	.981	0.004	.971	0.007	.036	-0.038	0.0264
	Strict	59.581	28	0.001	10.997	8	0.202	.979	0.000	.977	-0.002	.032	0.006	0.0247
Nationality	Configural	33.681	14	0.002				.986		.971		0.037		0.0266
	Metric	49.064	19	0.001	15.383	5	0.009	.979	-0.001	.967	-0.002	.039	0.019	0.0277
	Strong	49.107	20	0.001	0.043	1	0.835	.980	0.004	.970	0.007	.037	-0.038	0.0281
	Strict	62.615	28	0.001	13.508	8	0.096	.976	0.000	.974	-0.002	.034	0.006	0.0289
Major	Configural	99.142	56	0.001				.971		.969		0.026		0.0809
	Metric	105.102	61	0.001	5.959	3	0.310	.971	-0.001	.971	-0.002	.025	0.019	0.0786
	Strong	105.400	62	0.001	0.298	1	0.585	.971	0.004	.972	0.007	.025	-0.038	0.0800
	Strict	114.107	70	0.001	8.707	8	0.368	.971	0.000	.975	-0.002	.024	0.006	0.0834
Academic Grade	Configural	91.488	56	0.002				.976		.974		0.024		0.0401
	Metric	95.492	61	0.003	4.004	5	0.549	.977	-0.001	.977	-0.002	.023	0.019	0.0397
	Strong	96.247	62	0.003	0.755	1	0.385	.977	0.004	.978	0.007	.022	-0.038	0.0371
	Strict	104.816	70	0.004	8.569	8	0.380	.977	0.000	.980	-0.002	.021	0.006	0.0359
Internet activities	Configural	100.552	56	0.001				.970		.968		0.027		0.0398
	Metric	104.373	61	0.001	3.821	5	0.575	.971	-0.001	.971	-0.002	.025	0.019	0.0434
	Strong	104.466	62	0.001	0.093	1	0.760	.971	0.004	.972	0.007	.025	-0.038	0.0425
	Strict	109.621	70	0.002	5.156	8	0.741	.973	0.000	.977	-0.002	.023	0.006	0.0401

χ^2: chi-square; df: degrees of freedom; CFI: comparative fit index; TLI: Tucker–Lewis index; RMSEA: root mean square error of approximation; CI: confidence interval; SRMR: standardized root mean residual
