# Supplementary material for: The Six-Item Version of the Internet Addiction Test: Its Development, Psychometric Properties, and Measurement Invariance among Women with Eating Disorders and Healthy School and University Students
Source: Int J Environ Res Public Health. 2021 Nov 24;18(23):12341. doi: 10.3390/ijerph182312341 (PMC8657305; doi:10.3390/ijerph182312341)
Supplement: Supplementary file 1 [file ijerph-18-12341-s001.zip › Supplementary Table S4.pdf]

3.2.2. Factor strcuture of the Internet Addiction Test as revealed by EFA in the EDs sample

Supplementary Table S4. Summary of exploratory factor analysis (maximum likelihood with Varimax rotation) of the Spanish version of the Internet Addiction Test.

**Criteria**	**Whole sample (N = 123)**	**Anorexia nervosa (N = 59)**	**Other eating disorders (N = 64)**
Range of item communalities	0.788 – 0.947	0.670 – 0.948	0.857 – 0.977
Percentage of variance explained	83.4%	74.4%	87.4%
Kaiser–Meyer–Olkin test	0.956	0.910	0.942
Bartlett's test χ^2 (df)	4172.9 (190) **	1562.8 (190) **	2416.4 (190) **

χ^2: chi square test, df: degree of freedom, **: p = 0.000.

EFA resulted in a single factor explaining 74% - 87% of the total variances in the samples. All items had high commonalities while high values of KMO test and significant χ^2 of Bartlett's test indicated appropriateness of the participant-to-item ratio and sample size for an EFA in the samples (Table 2)—further details in the Supplementary material (e.g., the scree plots, item communalities, etc.).
